# Expression of Antimicrobial Peptides and Cytokines in Human Omentum Following Abdominal Surgery

**DOI:** 10.7759/cureus.17477

**Published:** 2021-08-26

**Authors:** Meenu Srivastava, Abhijit Chandra, Rahul R, Jaya Nigam, Pritheesh Rajan, Devendra Parmar, Rajeshwar N Srivastava, Vivek Gupta

**Affiliations:** 1 Surgical Gastroenterology, King George's Medical University, Lucknow, IND; 2 Surgical Gastroenterology, Sanjay Gandhi Postgraduate Institute of Medical Sciences, Lucknow, IND; 3 Developmental Toxicology, Indian Institute of Toxicology Research, Lucknow, IND; 4 Orthopaedic Surgery, King George's Medical University, Lucknow, IND

**Keywords:** omentum, antimicrobial peptide, immunoblotting, rt-pcr, abdominal surgery, cytokines

## Abstract

Introduction

Omentum can secrete out biological agents like different growth factors, cytokines, and antimicrobial peptides. The aim of our study was to determine the expression of antimicrobial peptides and cytokines in human omentum tissue and its response to intra-abdominal infection.

Methodology

Omentum tissue was obtained from 60 patients: control (n=20) and cases (n=40). mRNA expression of antimicrobial peptides (LL-37, HBD-1, HBD-2, HNP1-3) and cytokines (TNF- α, IL-8, IL-10, IL1β) was evaluated using Real-Time PCR. Protein quantification was done by Immunoblotting and ELISA.

Results

Significantly higher expression of antimicrobial peptides (LL-37, HBD-1, HBD-2, HNP1-3) and cytokines (TNF- α, IL-8, IL-10, IL1β) was observed in cases as compared to control at both the transcriptional and translational level (p<0.0001).

Conclusion

Omentum governs a population of antimicrobial peptides with potent immunologic functions. The expression of antimicrobial peptides and cytokines is inducible and increases with the severity of infection. Omentum is thus an immunologically active and adaptable organ but its complete regulatory mechanism is still elusive.

## Introduction

Wound infection following abdominal surgery is common and adds to morbidity and mortality. It increases the length of hospital stay as well as the cost of treatment. Apart from the use of modern aseptic techniques and wide range of powerful antibiotics, innate immunity has a pivotal role to play in the prevention and management of postoperative infection [1]. Omentum has been recognized as an integral part of the human immune system and physically acts as a barrier for infection, and is also referred to as “abdominal policeman”. It recognizes and adheres to the site of infection or injury and helps cordon off the site of inflammation, thus preventing the spread of infection. Omentum, in itself, is an organ that is responsible for the absorption and clearance of bacteria from the peritoneal cavity [2]. It comprises various cell types that include pre-adipocytes, adipocytes, stromal vascular cells, macrophages, and tissue matrix. It possesses the ability to secrete biological agents like antimicrobial peptides and cytokines to help in wound healing and control ongoing infection as demonstrated by us earlier [3].

Antimicrobial peptides (AMPs), also known as cationic host peptides, are present in all forms of life and are an important part of innate immunity [4]. AMPs can be divided into four groups based on amino acid composition and secondary structures: (i) α-helical peptides, (ii) β-sheet peptides stabilized by 2-4 disulphide bonds, (iii) extended structures, and (iv) loop peptide with one disulfide bond [5]. Defensin and cathelicidins are two AMPs which are the most extensively studied peptides of the mammalian gene family and their expression varies based on tissue type. Cathelicidin or LL-37 is a human cationic AMP of 18 kDa and is present in the epithelial cells of urogenital, respiratory, and gastrointestinal tracts, and is very effective against bacteria and fungi [6]. Defensins are categorized into α & β subgroups. HNP 1-3 belongs to the α defensin peptide family and HBD-1, HBD-2 belongs to the β defensin peptide family. AMPs like HBD-1, HNP1-4, and HD5-6 are constitutively expressed, while other AMPs like HBD2-4 and Bovine TAP show up-regulated expression during infection and inflammation [7].

Mesothelial cells in the omentum are known to enhance the production of cytokines such as TNF- α, IL-6, IL-1, and IL-8 in case of primary bacterial peritonitis [8]. Cytokines bind to extracellular matrix (ECM) protein or cell surface receptors which results in triggering a cascade of molecular events responsible for wound healing. [9]

We have previously demonstrated the expression of both AMPs and cytokines by human omentum that is inducible by lipopolysaccharides in vitro studies [3]. The aim of this study was to determine the expression of antimicrobial peptides (LL-37, HBD-1, HBD-2, HNP1-3) and cytokines (TNF- α, IL-8, IL-10, IL1 β) in omentum tissue in vivo. We also wanted to determine if there are any differences in their expression and in omentum tissue obtained from patients who have undergone abdominal surgery and/or suffered from intra-peritoneal infection.

## Materials and methods

Reagents used

TRIzol (Invitrogen, Thermo Fisher Scientific USA), BCA Protein Assay (Thermo Fisher Scientific,USA), Nanodrop (ND1000) Spectrophotometer (Thermo Fisher Scientific USA), cDNA Reverse Transcription Kit (Applied Biosystems, USA), Power SYBR Green PCR master mix (Applied Biosystems, USA) Enhanced chemifluorescence reagent (ECL Plus) from GE Healthcare USA. Tris-base solution (TBS), Tris/Glycine/SDS buffer and polyacrylamide gradient gels from Bio-Rad Laboratories (Hercules, CA). Polyvinylidene difluoride (PVDF) membranes from Immobilon-P, Millipore (Billerica, MA). Protease inhibitor from G-Biosciences /Genotech (St. Louis, MO).

Patients

Omentum tissue was obtained from the Department of Surgical Gastroenterology and Department of Surgery (accident and emergency) after approval (Letter No.5178/R-Cell-13) of the Institutional Ethics Committee of King George’s Medical University, India. Written informed consent was obtained from all the enrolled subjects. The study was performed between July 2015-January 2018.

Omentum tissue was obtained from 60 patients. Detailed history related to previous intervention, history suggestive of abdominal infection, hospitalization, use of prolonged antibiotics and co-morbidities were documented. Patients with history of diabetes and those with suspected or proven tuberculosis or malignancy were excluded from the study. The patients were divided into two groups-

Control group (naive omentum) (n=20) were obtained from the patients who underwent diagnostic laproscopy to rule out inflammatory condition, when no obvious inflammation, or stricture was noted, the final biopsies taken from the omentum were negative for any inflammatory pathology.

Cases group (activated omentum; n=40) included patients undergoing surgery as an elective procedure with or without previous history of abdominal infection or intervention or those with frank peritonitis.

Cases group were further divided into two subgroups: Case 1 (Clean-contaminated; n=20) and Case 2 (Contaminated; n=20).

Case 1: Patients with previous history of clean contaminated surgery (benign biliary stricture-08, complicated gall stone disease -08, chronic calcific pancreatitis -02, corrosive esophageal stricture -02).

Case 2: Patients operated in the emergency for generalized peritonitis or intra-abdominal contamination (perforation peritonitis-15, acute intestinal obstruction with gangrene-05).

Approximately 1-2 gm of omentum tissue was obtained at the start of surgery. Omentum tissue was divided into two parts. One part was taken in Trizol for RNA extraction and the other was snap frozen in liquid nitrogen for protein extraction. These tissue samples were stored at -80°C till further processing.

Sample preparation

Omentum was homogenized in Radio immune precipitation Assay (RIPA) buffer (0.1% SDS, 0.5% sodium deoxycholate, 1% Nonidet P-40, 150 mM NaCl, and 50 mM Tris- HCl, pH 8.0), supplemented with protease inhibitors. Cellular debris and lipids were removed by centrifugation of the solubilized samples at 13,000 rpm for 60 minutes recovering the soluble fraction below the fat containing supernatant, and preventing the non-homogenized material to settle at the bottom of the centrifuge tube. Protein concentration was determined by BCA Protein Assay.

Detection of (mRNA) of antimicrobial peptides (LL-37, HBD-1, HBD-2, HNP1-3) and cytokines (TNF-α, IL-1β, IL-8, IL10) in omentum tissue by Real-time PCR

Total RNA was isolated from naive and activated omentum tissue using TRIzol according to the manufacturer’s protocol. The purity of RNA was assessed by Nanodrop (ND1000) Spectrophotometer using A 260/280 ratio. cDNA synthesis was carried out with 1 µg of RNA using High-Capacity cDNA Reverse Transcription Kit. Real-time PCR assay was carried out using Power SYBR Green PCR master mix as earlier described by Chandra et al. [3]. Specific primer sequences for (HNP1-3, LL-37, HBD1, HBD2, TNF-α, IL-8, IL-10, IL-1β) [3] and β-actin [10] are in Supplementary Table 1.

β-actin (housekeeping gene) was used as an internal standard. The thermal cycling conditions consist of an initial step of 50ºC for 2 minutes, followed by denaturation at 95º C for 15 minutes. Further 45 cycles of denaturation (95ºC for 15 seconds) and annealing and extension step (60ºC for 60 seconds) were performed. PCR reactions were carried out in triplicate for each sample. Dissociation reaction was also carried out for each primer to check the specificity of primers. The comparative Ct method for relative quantification (ddCt method), which describes the change in expression of the target gene in a test sample relative to a calibrator sample, was used to analyze the data [11]. Data were analyzed using 7900HT Sequence Detector System (SDS) software version 2.2.1 (Applied Biosystems, USA).

Immunoblotting for antimicrobial peptides (LL-37, HBD-1, HBD-2, HNP1-3)

Immunoblotting was performed as described earlier in our study [12]. Protein extraction was done using RIPA lysis buffer (0.1% SDS, 0.5% sodium deoxycholate, 1% Nonidet P-40, 150 mM NaCl, and 50 mM Tris- HCl, pH 8.0), and 20 μg of protein per sample was resolved on 10-14% SDS PAGE followed by transfer onto a nitrocellulose membrane. The membrane was blocked with 5% (w/v) BSA in TBS buffer with 0.1% Tween 20 at room temperature for 60 minutes. Immunoblotting was performed with primary antibodies (1:2000 rabbit anti-human LL37(Cat.no.A1640) polyclonal antibody, 1:2000 rabbit anti-human HNP1-3 (Cat.no.AB37410) polyclonal antibody, and 1:2000 rabbit anti-human HBD2 (Cat.no.A1643) polyclonal antibody from Immunotag, Geno Technology, St. Louis, US and 1:2000 rabbit anti-human HBD1 (Cat.no.14738-1-AP) polyclonal antibody from Proteintech US, 1:2000 rabbit anti-human β-actin (Santa Cruz Biotechnology, USA)) at 40°C for overnight with gentle rocking. After the incubation with the primary antibody, the membranes were washed in TBS with 0.1% Tween 20 and incubated with the appropriate IgG-HRP-conjugated secondary antibody Novolink Min Polymer (Cat.no.RE-7290) Novocastra (Leica Biosystems, New Castle, USA) and detected by chemiluminescence reagent (ECL plus) (GE Healthcare USA). Immunoreactive bands were visualized by using the digital gel image analysis system (ImageQuant LAS 500) (GE Healthcare Biosciences Sweden). Optical densities of the immune reactive bands were measured using Alpha Ease FC software (Alpha Innotech, Santa Clara, CA, USA).

Enzyme-linked immunosorbent assay for cytokines (TNF-α, IL-1β, IL-8, IL10)

Protein levels of the cytokines TNF-α (Cat.no. ELH-TNF- α-CL), IL-8 (Cat.no. ELH-IL8-CL), IL10 (Cat.no. ELH-IL10-CL) (Ray Biotech Life Science Inc, Cloud Clone Corp, Houston, TX), and IL-1β (Cat.no. CEK1731) Bioworld Technology, Inc. (USA) were measured in the tissue homogenates using commercially available enzyme-linked immunosorbent assay (ELISA) Kit as per the manufacturer’s protocol.

Statistical analysis

Statistical analysis was done using GraphPad Prism version 5.0 (GraphPad Software, Inc., California, USA). Multiple groups were analyzed using one-way analysis of variance (ANOVA) followed by Tukey post hoc test. A P-value <0.005 was considered statistically significant.

## Results

The age (mean±S.E) of the subjects enrolled in the study was 36.33±3.6 years (control), 37.33±3.02 years (Case 1), and 35.73±2.57 years (Case 2), and the age range was 20-60 years. In the control group, 9 (45%) males and 11(55%) females were enrolled, while in Case 1, 7 (35%) males and 13 (65%) females and in Case 2, 8 (40%) males and 12 (60%) females were enrolled.

mRNA levels of AMPs and cytokines in omentum tissue

**Figure 1:**
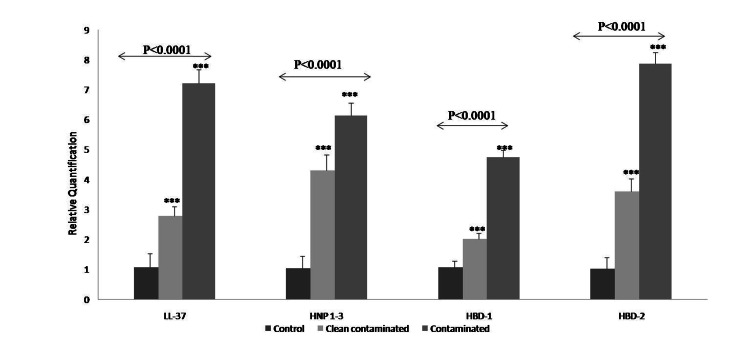
mRNA expression of antimicrobial peptide in omentum Altered expression of mRNA AMP (LL-37, HNP1-3, HBD-1, HBD-2) genes in clean-contaminated and contaminated omentum tissue as compared to control / naïve omentum tissue. RT PCR was performed in triplicate by 2x Power SYBR Green PCR master mix. β-Actin was used as an internal control to normalize the data. mRNA expression is expressed in fold change.

**Figure 2:**
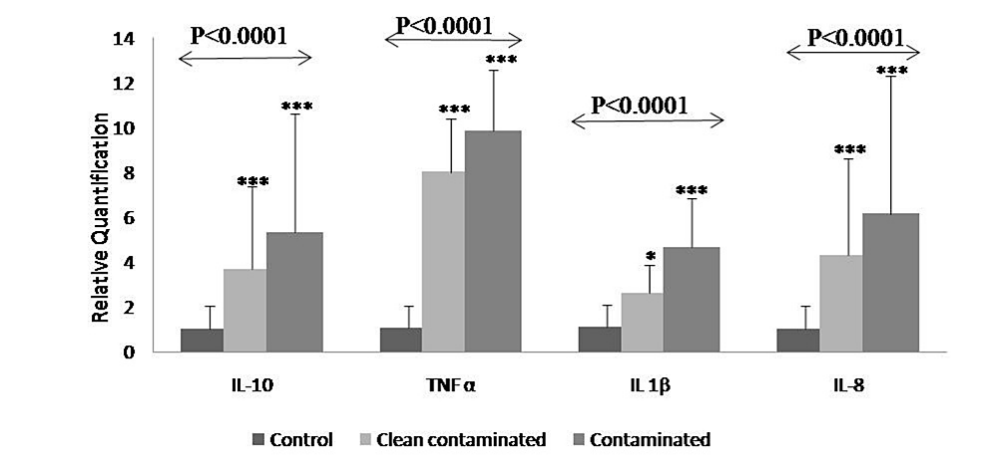
mRNA expression of cytokines in omentum Data is represented as relative quantification ± standard error (RQ ± SE.) obtained through real-time PCR. The statistical difference between the samples was analyzed by  test (ANOVA and Tukey’s post hoc test) * Indicates statistically significant difference (P <0.05) ** Indicates statistically significant difference (P<0.01) *** Indicates statistically significant difference (P<0.001)

mRNA expression of AMPs and cytokines were analyzed in omentum tissue of control and cases. Significantly higher expression of AMPs (P˂0.0001) (Figure 1, Table 1) and cytokines (P˂0.0001) (Figure 2, Table 2) was observed in contaminated (Case 2) as compared to clean contaminated (Case 1) and Control group. Amongst the AMP, maximum expression was observed in HBD-2, while the least expression was shown by HBD-1 in the contaminated (Case 2) group. In cytokines, TNF-α was seen to have the maximum surge.

**Table 1:** mRNA expression of antimicrobial peptide in omentum Data is represented as relative quantification ± standard error (RQ ± SE.) obtained through real-time PCR. The statistical difference between the samples was analyzed by  test (ANOVA and Tukey’s post hoc test) *** Indicates statistically significant difference (P<0.001)

Genes	Control (RQ± SE.)	Clean-Contaminated (RQ± SE.)	Contaminated (RQ± SE.)	p-value	Tukey’s multiple comparison
Antimicrobial Peptides					
LL-37	1.06±0.55	2.78±0.31	7.21±0.45	<0.0001	Control Vs Clean-contaminated *** ControlVsContaminated*** Clean-contaminated Vs Contaminated ***
HNP 1-3	1.03±0.11	4.31±0.51	6.14±0.41	<0.0001	Control Vs Clean-contaminated *** ControlVsContaminated*** Clean-contaminated Vs Contaminated ***
HBD -1	1.06±0.32	2.02±0.19	4.75±0.22	<0.0001	Control Vs Clean-contaminated *** Control Vs Contaminated*** Clean-contaminated Vs Contaminated ***
HBD -2	1.02±0.23	3.60±0.43	7.87±0.37	<0.0001	Control Vs Clean-contaminated *** Control Vs Contaminated*** Clean-contaminated Vs Contaminated ***

**Table 2:** mRNA expression of cytokines in omentum Data is represented as relative quantification ± standard error (RQ ± SE.) obtained through real-time PCR. The statistical difference between the samples was analyzed by  test (ANOVA and Tukey’s post hoc test) * Indicates statistically significant difference (P <0.05) ** Indicates statistically significant difference (P<0.01) *** Indicates statistically significant difference (P<0.001)

Genes	Control (RQ± SE.)	Clean-Contaminated (RQ± SE.)	Contaminated (RQ± SE.)	p-value	Tukey’s multiple comparison
Cytokines					
IL-10	1.02±1.24	3.68±1.56	5.31±2.31	P<0.0001	Control Vs Clean-contaminated *** Control Vs Contaminated*** Clean-contaminated Vs Contaminated **
TNF-α	1.06± 0.96	8.0±2.39	9.84±2.72	<0.0001	Control Vs Clean-contaminated *** Control Vs Contaminated*** Clean-contaminated Vs Contaminated *
IL-8	1.03±0.11	4.31±0.51	6.14±2.34	P<0.0001	Control Vs Clean-contaminated *** Control Vs Contaminated*** Clean-contaminated Vs Contaminated *
IL-1β	1.09±0.86	2.60±1.24	4.65±2.19	P<0.0001	Control Vs Clean-contaminated ** Control Vs Contaminated*** Clean-contaminated Vs Contaminated *

Protein expression of AMPs and cytokines

**Figure 3:**
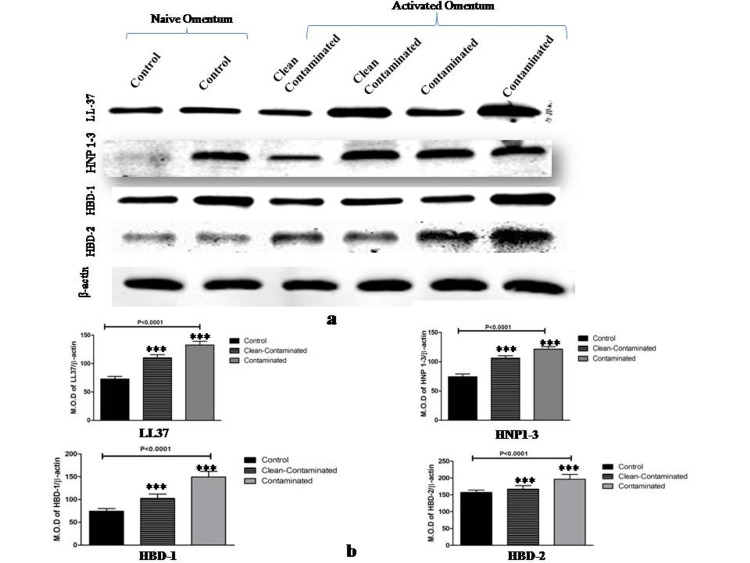
Immunoblot detection of LL-37, HNP1-3, HBD-1, HBD-2 proteins in Omentum Tissue. (a) Immunoblot detection of anti-microbial proteins (LL-37, HNP1-3, HBD-1, HBD-2) and β-actin in clean-contaminated and contaminated omentum tissue as compared to control / naïve omentum tissue. (b) Densitometric analysis of Immunoblots for anti-microbial proteins (LL-37, HNP1-3, HBD-1, HBD-2) in clean-contaminated and contaminated omentum tissue as compared to control / naïve omentum tissue. β-Actin was used as an internal control to normalize the data. All the values represent mean± S.D (*p<0.05-significant, ***p<0.0001-highly significant). MOD-Mean Optical Density

Immunoblot demonstrated the presence of AMPs (LL-37, HNP1-3, HBD1, and HBD-2) in omentum samples (control and cases). In line with mRNA expression, protein levels of LL-37 (P<0.0001), HNP1-3 (P<0.0001), HBD2 (P<0.0001), and HBD1 (P<0.0001) were significantly higher in cases (both case 1 and 2) as compared to control. Amongst all AMPs, maximum expression was observed in HBD-2 in the Contaminated group (Case 2) (Figure 3a, 3b).

**Figure 4:**
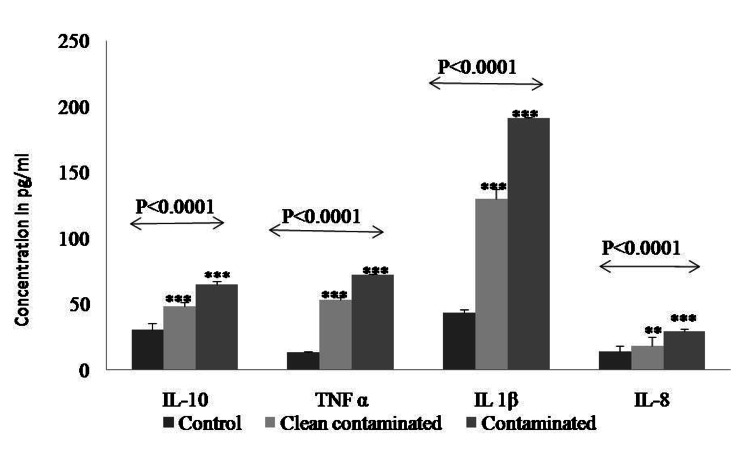
Protein expression of cytokines by ELISA. Alterations in the activity of cytokines in clean-contaminated and contaminated omentum tissue through ELISA kit. Data were analyzed in pg/ml through preparation of standard curve as supplied by manufacturer.

Significantly higher protein expression (P < 0.0001) of cytokines was detected in the Contaminated group (Case 2) as compared to the clean-contaminated (case 1) and control group by ELISA. IL-1β expression was highest among all cytokines in contaminated group (Figure 4, Table 3).

**Table 3:** Protein expression of cytokines by ELISA. Data are represented as mean ± standard error (SE). The statistical difference between the samples was analyzed by  test (ANOVA and Tukey’s post hoc test) ** Indicates statistically significant difference (P<0.01) *** Indicates statistically significant difference (P<0.001)

Genes	Contro (Mean± SE.)	Clean-Contaminated (Mean± SE.)	Contaminated (Mean ± SE.)	p-value	
TNF-α	13.40±0.52	53.11±1.7	72.01± 0.6	<0.0001	Control Vs Clean-contaminated *** Control Vs Contaminated*** Clean-contaminated Vs Contaminated ***
Range (pg/ml)	6.1-56	9.3-88	11.01-96		
IL-8	14.07±4.5	18.52±6.3	29.51 ± 1.6	<0.0001	Control Vs Clean-contaminated ** Control Vs Contaminated*** Clean-contaminated Vs Contaminated ***
Range (pg/ml)	2.5-33	7.5-54	5.3 -76		
IL-10	30.77±4.52	48.5±2.97	65.03 ±2.3	<0.0001	Control Vs Clean-contaminated *** Control Vs Contaminated*** Clean-contaminated Vs Contaminated ***
Range (pg/ml)	18.75-58	36-67	43-81		
IL-1β	43.5±2.5	130±7.5	191.0±1.09	<0.0001	Control Vs Clean-contaminated *** Control Vs Contaminated*** Clean-contaminated Vs Contaminated ***
Range (pg/ml)	30-56	90-156	70-183		

## Discussion

Abdominal and surgical site infections (SSI) are a major problem in hospitals despite the advances in asepsis, sterilization, antimicrobial drugs, and operative techniques [13]. The severity of such infections varies with the type of surgery, the integrity of host defense, and the bacterial load. Omentum is an integral part of the immune system in the abdomen and its wound healing properties are well known [14]. It is reported that omentum has a role in decreasing the infection rate in aortic graft implantation and sealing of intestinal perforation in neonates [15]. We have shown that human omentum exhibits an antimicrobial spectrum of its own with a definite antibiotic sensitivity pattern [12]. Other studies have also shown the decline of inflammation, as the omentum adheres to and wards off the area of insult [2]. However, the physiology behind the immune functions of the omentum is not completely understood.

On exposure to foreign substances, omental blood flow increases and releases inflammatory, chemotactic, and homeostatic substances from its expanded stromal tissue [16]. Our study has also demonstrated high expression of cytokines and AMP at both transcription and translational levels in the activated omentum patient group, particularly in presence of peritonitis.

Antimicrobial peptides are produced by a wide variety of organisms and these biologically active molecules are an essential component of their innate immune response [17]. AMPs, through host defense mechanism, kill the invading pathogenic micro-organisms and acts as an immune modulator in higher organisms [18]. They are known to possess broad-spectrum antimicrobial activity with minimal resistance due to their diverse structures and sequence. In mammals, AMPs can be categorized into two major groups: cathelicidins and defensins [19]. Both cathelicidins and defensins are secreted at the site of infection or injury. They induce the transcription and secretion of chemokine and induce histamine release from mast cells. This leads further to the recruitment of innate and adaptive immune cells required for the cellular and humoral response to a pathogen [20].

In chronic inflammatory diseases like psoriasis, cystic fibrosis, and bronchiolitis, significantly increased expression of defensins and cathelicidins have been reported [21-22]. In active inflammatory bowel disease, increased expression of HNP1-3 from intestinal epithelial cells has also been reported [23]. A prospective cross-sectional study investigated the systemic release of endogenous HNP1-3 in children with severe sepsis. Patients with sepsis demonstrated higher expression of HNP1-3 as compared to non-septic critically ill patients [24]. Several other case-control studies have also demonstrated higher expression of AMPs in patients with severe sepsis [25-26]. In another study by Lippross et al., in patients with multiple traumatic injuries, the bacterial infection rate was found to be surprisingly low due to elevated levels of AMP following trauma [27].

We have also previously demonstrated the antimicrobial and anti-inflammatory properties of human omentum. In this in vitro study, we had demonstrated that the human omentum adipocytes consistently express antimicrobial peptides (LL-37, HNP 1-3, HBD-1 and HBD-2) and cytokines (IL-1b, IL-2, IL-4, IL-8, IL-10, TNF-α, and GM-CSF) in a dose-dependent manner to increasing concentrations of LPS, mimicking experimental sepsis and the extent of its severity. This pattern is reflected in our present in vivo study as well. Consistent with our initial study, the expression of cationic host peptides (LL-37, HNP1-3, HBD-1, HBD-2) was markedly high in the contaminated group wherein omentum was exposed to severe inflammation and gross contamination, and significantly lower in the control group (with no exposure to the inflammatory process). The expression of AMPs increased both at transcription and translation levels correlating with the severity of infection which reflects the ability of human omentum to adapt and resist changes to its infectious environment. The expression of HBD-2, both at mRNA and protein levels, is relatively more as compared to HBD-1, a trend found in our previous study as well [12] signifying its inducing expression to increasing infection.

Cytokines act as regulatory molecules in adipose tissue metabolism. Research-based evidence suggests that peritoneal macrophages are activated during abdominal surgery and release pro-inflammatory cytokines (IL-1, TNF- α, IL-6) into the peritoneal fluid in the abdominal cavity. They play a major role in wound healing [28-29]. Bassols et al. reported that human omentum tissue produces pro-inflammatory cytokines and chemokines when exposed to infection [30]. We have also previously shown that many of these cytokines (IL-1b, IL-2, IL-4, IL- 8, IL-10, TNF-a, and GM-CSF) can be experimentally induced by LPS in human omental adipocytes [3]. Our present clinical study has also shown that the expressions of cytokines TNF- α, IL- 1β, IL-8, and IL-10 at mRNA and protein levels were significantly higher in contaminated and clean-contaminated groups (activated omentum) as compared to the controls (P<0.0001). This indicates that the human omentum is immunologically active and has the ability to combat infection by releasing cytokines during an inflammatory process. Cytokine secretion by omentum is also inducible and varies with the severity of infection and extent of inflammation.

Few limitations of the study are firstly the sample size. We have a limited number of samples in the control group due to the unavailability of patients for diagnostic laparoscopy. Secondly, we studied only the selected panel of antimicrobial peptides and cytokines.

## Conclusions

The present study provides new insights into the molecular mechanism of the differential immune and antimicrobial activity of the human omentum in the different clinical settings that represent the varying degrees of intra-abdominal infection. The activated omental tissue releases a panel of AMPs and cytokines that have potent immunologic, anti-inflammatory, antimicrobial functions, the expression of which increases with the severity of peritoneal infection. Omentum is, therefore, an immunologically active organ with the ability to actively adapt to the changes in its environment. However, the mechanism involved in the immune function of the omentum needs further exploration.

## Appendices

**Table 4:** Specific primer sequences

Gene Name	Primer Sequence
LL-37	f: 5’-GAAGACCCAAAGGAATGGCC-3’ r: 5’-CAGAGCCCAGAAGCCTGAGC-3’
HNP1-3	f: 5’- CTTGGCTCCAAAGCATCC-3’ r : 5’-GAACCTGCATCTACCAGGGA-3’
HBD-1	f:5’-CCCAGTTCCTGAAATCCTGA-3’ r: 5’-CAGGTGCCTTGAATTTTGGT-3’;
HBD-2	f: 5’-CCAGCCATCAGCCATGAGGGT-3’ r: 5’-GGAGCCCTTTCTGAATCCGCA-3’.
TNF α	f: 5’-CCCAGGCAGTCAGATCATCTTC-3’ r: 5’-AGCTGCCCCTCAGCTTGA-3’
IL8	f: 5’-ATGACTTCCAAGCTGGCCGTGGCT-3’ r: 5’-TCTCAGCCCTCTTCAAAAACTTCTC-3’
IL1β	f: 5’-ACAGATGAAGTGCTCCTTCCA-3’ r: 5’-GTCGGAGATTCGTAGCTGGAT-3’
IL-10	f: 5’-GCCGTGGAGCAGGTGAAG-3’ r: 5’-GAAGATGTCAAACTCACTCATGGCT-3’
β-actin	f: 5’-ACGCAGACATCGTCATCC-3’ r:5’-AACCGAGTTGAACCACG-3’
